# Static and Dynamic Performance of Steel-Fiber-Reinforced Polymer-Modified Concrete: Strength, Toughness and Crack Resistance

**DOI:** 10.3390/ma19183813

**Published:** 2026-09-08

**Authors:** Zhixiang Wang, Zhijian Yi, Ya Li, Jiaming Zhang, Kang Su, Jie Liu

**Affiliations:** School of Civil Engineering, Chongqing Jiaotong University, No. 66, Xuefu Road, Nan’an District, Chongqing 400074, China

**Keywords:** polymer-modified concrete, ultrashort ultrafine steel fibers, flexural performance, post-cracking load-carrying capacity, microstructure

## Abstract

Conventional concrete pavement materials remain limited in flexural strength, deformability, post-cracking load-carrying capacity, and impact resistance. To address these deficiencies, this study investigates the effects of polymer modification and ultrashort ultrafine steel fiber reinforcement on the static and dynamic mechanical responses and crack evolution of concrete. The results show that polymer modification enhances deformability, while steel fiber incorporation further increases flexural strength and ultimate flexural strain and improves post-cracking load-carrying capacity. Under repeated impact loading, the nominal impact energy input required for initial cracking and final failure increased; in particular, polymer-modified concrete containing 5% steel fibers showed increases of 938.76% in ultimate flexural strain and 8682.63% in the number of impacts to failure relative to ordinary concrete. The matrix and interfacial morphologies observed by scanning electron microscopy (SEM) were consistent with the macroscopic mechanical responses, supporting the interpretation that polymer modification improves matrix and interfacial integrity, while steel fibers contribute to post-cracking load transfer through crack bridging. Overall, the material exhibited high deformability and damage tolerance, indicating its potential for specialized pavement applications.

## 1. Introduction

Conventional concrete pavement materials commonly suffer from relatively low flexural strength, limited deformability, pronounced brittleness, and insufficient post-cracking load-carrying capacity. To address the combined requirements of pavement materials for strength and deformation performance, our research group developed polymer skeleton porous concrete, which exhibits both relatively high flexural strength and substantial deformation capacity and has been explored in engineering applications [1,2,3]. As its application has expanded to specialized pavement scenarios, higher requirements have been placed on flexural strength, sustained post-cracking load-carrying capacity, and impact resistance. Accordingly, steel fibers were incorporated into the original polymer skeleton porous concrete to develop steel-fiber-reinforced polymer-modified concrete [4,5,6], with the aim of improving its flexural performance, deformability, and impact resistance and thereby enhancing its adaptability to the performance requirements of specialized pavement applications. The engineering application of this material has also been explored in the steel bridge-deck pavement of the Mafang Bridge in Guangdong, China.

In recent years, steel-fiber-reinforced polymer concrete and related fiber-reinforced composites have been extensively investigated in different countries and regions, although the research emphasis has varied considerably. In China, research has focused primarily on the combined effects of polymer emulsions and steel fibers on the macroscopic mechanical properties and microstructure of cementitious materials. Xia et al. investigated recycled aggregate concrete modified with waterborne epoxy resin and steel fibers and reported that steel fiber incorporation markedly improved splitting tensile strength and repeated-impact resistance [7]. Li et al. examined the size effect of polymer-modified steel-fiber-reinforced cement mortar through flexural testing and mesoscale simulation, showing that specimen size affected both flexural strength and damage evolution [8,9]. European studies have focused more strongly on material composition, fiber-related effects, and the factors governing post-cracking performance [10,11,12,13]. Asteris et al., using polyester–resin polymer concrete, found that steel fibers had relatively limited effects on compressive strength and elastic modulus but substantially improved splitting tensile strength, flexural strength, and toughness [14]. Aidarov et al. further reported that the spatial orientation of steel fibers significantly affected flexural and post-cracking responses, indicating that the efficiency of fiber reinforcement is also constrained by the internal distribution of fibers [15]. Research in North America has placed greater emphasis on polymer matrix composition, post-cracking toughness, and structural applications [16,17,18,19]. Farooq and Banthia varied epoxy-resin content and curing conditions in fiber-reinforced polymer concrete and found that high-strength steel microfibers could induce pronounced tensile ductility and strain-hardening behavior, while polymer content and curing conditions also influenced the post-cracking response [20]. Kafaji and Azzawi tested steel-fiber-reinforced concrete bridge deck slabs and showed that steel fibers increased load-carrying capacity and reduced crack width, indicating their effectiveness in controlling cracking in flexural members such as bridge decks [21]. Despite differences in material systems and evaluation methods across regions, these studies consistently indicate that polymer modification primarily alters the state of the matrix and interfaces, whereas steel fibers enhance deformability and damage tolerance through crack bridging and post-cracking load transfer.

However, existing studies have mainly focused on specific polymer systems or individual properties, such as static strength, fracture behavior, or impact resistance [22,23,24,25,26,27]. Comprehensive evaluations that simultaneously consider strength, deformability, sustained post-cracking load-carrying capacity, and repeated-impact performance for specialized pavement applications remain relatively limited. To address this limitation, the present study investigates the static and dynamic mechanical responses and post-cracking behavior of concrete modified with polymer and ultrashort ultrafine steel fibers through compressive, flexural, and repeated drop-weight impact tests. Scanning electron microscopy (SEM) is further used to observe and analyze the matrix and interfacial morphological features.

## 2. Experimental Materials and Methods

### 2.1. Raw Materials

**Figure 1:**
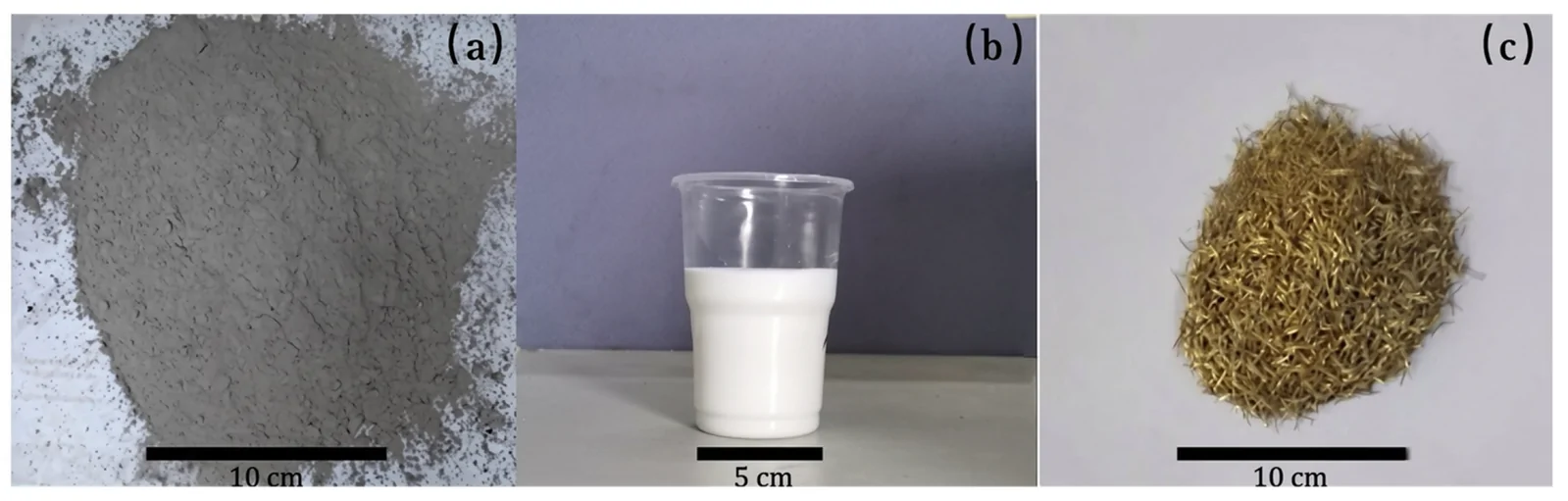
**Raw materials used in this study:** (**a**) cement; (**b**) specially formulated polymer emulsion; (**c**) ultrashort ultrafine steel fibers.

The raw materials used in this study are shown in Figure 1. P·O 42.5R Portland cement was used as the cementitious material. The polymer emulsion used in this study was an anionic modified polyacrylate emulsion developed by our research group. It was a white aqueous emulsion with a solid content of 38%, a pH of 7–9, a density of 1.02–1.06 g/cm^3^, and a viscosity of 100–1000 mPa·s [28]. The properties of the steel fibers are listed in Table 1. Crushed limestone with a particle size of 5–10 mm and a nominal maximum aggregate size of 10 mm was used as the coarse aggregate. Yellow sand was used as the fine aggregate. Its particle-size distribution was determined by sieve analysis. The dried sand sample was successively passed through standard sieves with aperture sizes of 9.50, 4.75, 2.36, 1.18, 0.60, 0.30, 0.150, and 0.075 mm. The cumulative passing percentage was calculated from the mass retained on each sieve, and the resulting particle-size distribution is presented in Table 2.

### 2.2. Mix Proportions

The base mix proportion used in this study was cement:sand:coarse aggregate:emulsion (water) = 1:1.10:1.79:0.52. For the polymer-modified concrete, the mixing water was replaced by an equal amount of polymer emulsion. Steel fibers were incorporated at volume fractions of 2% and 5%. The mix proportions and corresponding designations are listed in Table 3, where P, J, and C denote ordinary concrete, polymer-modified concrete, and ultrashort ultrafine steel fibers, respectively.

### 2.3. Specimen Preparation and Curing

To reduce steel-fiber agglomeration, the mixtures were prepared in stages using a forced-action mixer. Cement and aggregates were first dry-mixed, after which the steel fibers were added slowly in batches under continuous mixing. Once the fibers were uniformly dispersed, the polymer emulsion was added, and mixing was continued until a uniform mixture was obtained. The fresh mixture was then placed into molds, vibrated for compaction, and finished to a smooth surface.

Ordinary concrete and polymer-modified concrete were cured using regimes appropriate to their respective strength-development mechanisms [29,30,31]. Ordinary concrete relies primarily on continued cement hydration and was therefore subjected to standard moist curing. For polymer-modified concrete, both cement hydration and polymer film formation need to be considered, while prolonged exposure to high humidity is unfavorable for water loss and coalescence of the emulsion particles. Accordingly, the specimens were covered with plastic film for moisture retention for 24 h after casting, demolded, and then cured in ambient air at room temperature until the specified testing age. Because these two types of concrete are generally cured under conditions suited to their respective material characteristics in practical engineering, their mechanical properties were likewise evaluated under their corresponding curing regimes in this study. Accordingly, the differences between the two materials should be interpreted as their overall responses under their respective curing regimes rather than as effects of material composition alone.

### 2.4. Test Methods

#### 2.4.1. Compressive and Flexural Tests

**Figure 2:**
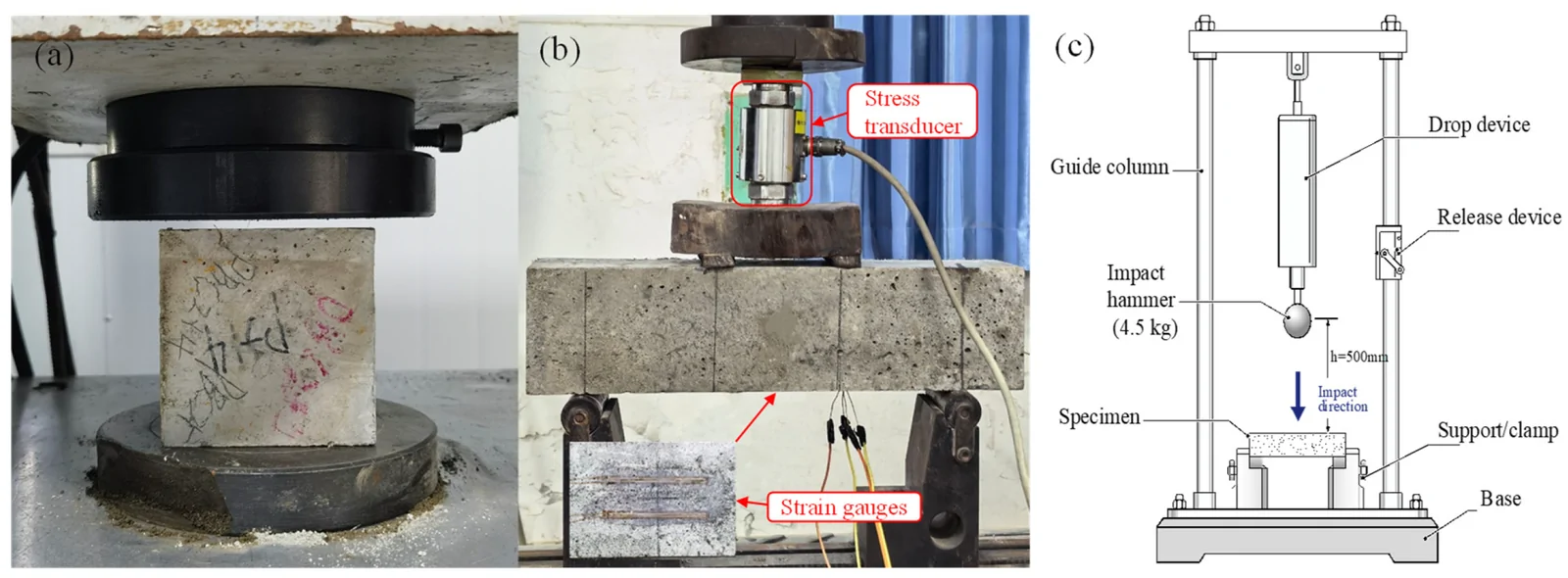
**Test setups:** (**a**) compressive test; (**b**) flexural test; (**c**) drop-weight impact test.

The compressive and flexural tests were conducted in accordance with the *Test Methods of Cement and Concrete for Highway Engineering* (JTG 3420—2020) [32]. Cubic specimens measuring 100 mm × 100 mm × 100 mm were used for the compressive test and loaded using a fully automatic compression testing machine at a loading rate of 5 kN/s, as shown in Figure 2a. Prismatic specimens measuring 100 mm × 100 mm × 400 mm were subjected to four-point bending using an MTS universal testing machine at a loading rate of 0.05 MPa/s, as shown in Figure 2b.

To measure the tensile strain response at the bottom of the specimen during flexural loading, two strain gauges with a gauge length of 80 mm were symmetrically bonded to the bottom surface at midspan. Strain data were recorded using a YE2539 high-speed static strain acquisition system with a strain resolution of 1 με and a sampling frequency of 1 Hz. The average reading of the two strain gauges at each time point was taken as the corresponding flexural strain, and the strain corresponding to the peak load was defined as the ultimate flexural strain. The displacement reported in the load–displacement curves refers to the vertical displacement of the loading head of the testing machine.

The compressive and flexural tests were conducted at curing ages of 3, 7, and 28 d. For each material group, three independent replicate specimens were tested at each age (n = 3). The arithmetic mean of the three replicate measurements was reported for each property, and the standard deviation was used to characterize data variability. For the load–displacement and flexural stress–strain curves, one specimen whose peak response and overall response characteristics were close to the group-average behavior was selected as the representative curve to avoid obscuring the figure with multiple overlapping curves. All statistical results for the reported performance indicators were calculated using data from all replicate specimens.

#### 2.4.2. Drop-Weight Impact Test

The drop-weight impact test was conducted in accordance with the *Standard Test Methods for Fiber Reinforced Concrete* (CECS 13:2009) [33], as shown in Figure 2c. Cylindrical specimens with a diameter of 150 mm and a height of 63 ± 3 mm were used. The drop hammer had a mass of 4.50 kg and a drop height of 500 mm, and each free fall of the hammer was counted as one impact cycle. After each impact, crack development on the top and bottom surfaces of the specimen was examined. The cumulative number of impacts at which the first visible crack appeared on the specimen surface was defined as the initial-cracking impact number. Impact loading was then continued until the specimen came into contact with any three of the four positioning lugs of the impact apparatus, and the corresponding cumulative number of impacts was defined as the failure impact number. The difference between the failure and initial-cracking impact numbers was used to represent the additional number of impacts sustained by the specimen from initial cracking to failure.

To compare the repeated-impact performance of the different specimen groups on an energy basis, the gravitational potential energy of the drop hammer for a single drop, mgh, was defined as the nominal impact energy per impact, representing the theoretical impact energy supplied by one impact. The nominal impact energy at initial cracking $W_{1}$, nominal impact energy at failure $W_{2}$, and post-cracking nominal impact energy reserve ∆W were calculated according to Equations (1)–(3), respectively:

(1) $$W_{1}=N_{1}mgh$$

(2) $$W_{2}=N_{2}mgh$$

(3) $$∆W=W_{2}-W_{1}=\left(N_{2}-N_{1}\right)mgh$$

where $N_{1}$ and $N_{2}$ are the cumulative numbers of impacts at initial cracking and failure, respectively; *m*, *g*, and *h* are the mass of the drop hammer, gravitational acceleration, and drop height, respectively. $W_{1}$, $W_{2}$, and ∆W are nominal impact input energies cumulatively calculated from mgh and are used to characterize the nominal impact-energy levels corresponding to initial cracking, failure, and the post-cracking stage, respectively [7,34,35,36]. These quantities do not represent the actual dynamic energy absorbed or dissipated by the specimens.

The drop-weight impact tests were conducted at 28 d. Considering the relatively high variability inherent in drop-weight impact test results, five independent replicate specimens were tested for each group. During data processing, the maximum and minimum values were excluded, and the arithmetic mean of the remaining three results was reported as the measured value for each group. The standard deviation was used to characterize data variability.

#### 2.4.3. Scanning Electron Microscopy (SEM)

Specimens cured for 28 d were immersed in ethanol, dried, and gold-coated before their microstructural morphology was examined using a KYKY-2800B scanning electron microscope (Beijing Zhongke Keyi Co., Ltd., Beijing, China) at an accelerating voltage of 20 kV.

## 3. Results and Discussion

### 3.1. Flexural Strength, Deformation Capacity, and Post-Cracking Load-Carrying Capacity

**Figure 3:**
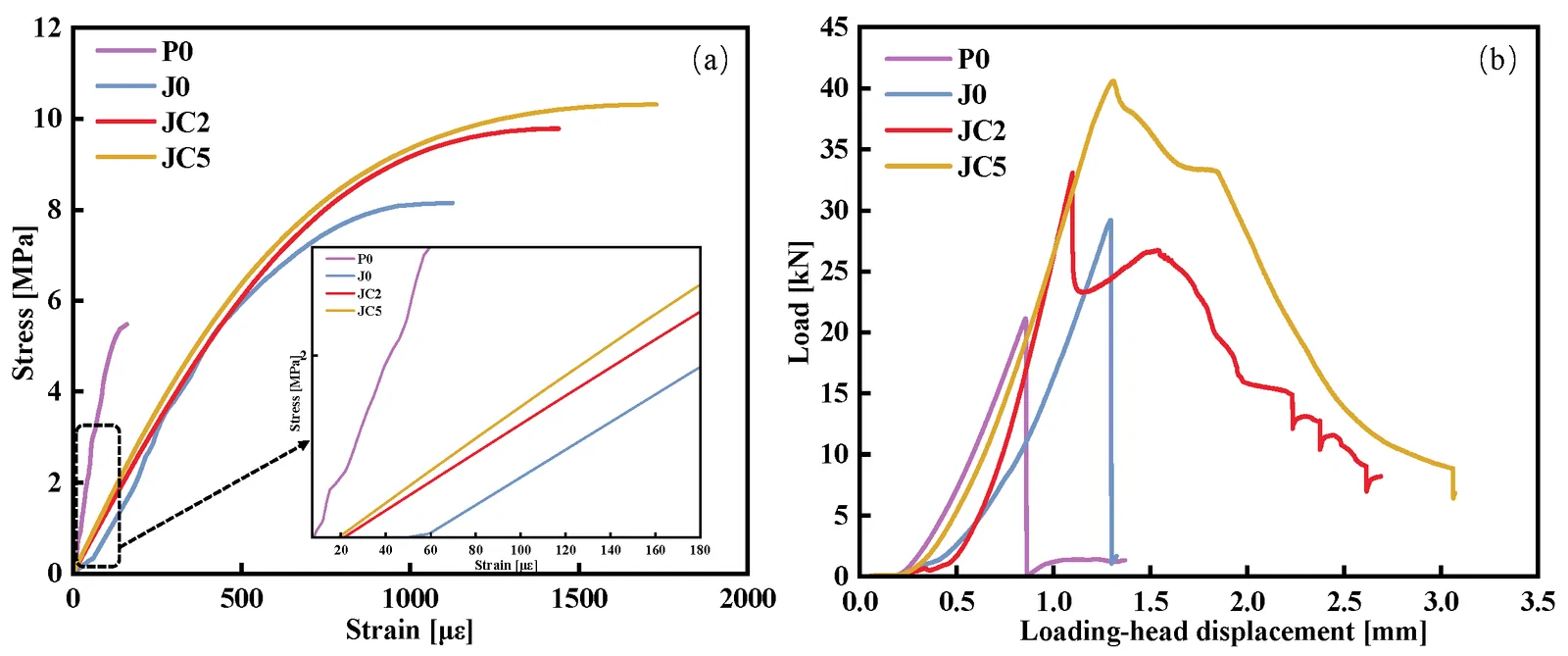
(**a**) Representative stress–strain curves; (**b**) representative load–displacement curves.

For pavement and bridge-deck paving materials, relatively high flexural strength and large deformation capacity are beneficial for resisting flexural stresses induced by repeated vehicle loading and accommodating warping deformation caused by temperature and moisture gradients. These properties can delay crack initiation and propagation, reduce the risk of premature cracking, and improve service durability [37,38]. However, conventional pavement materials often have difficulty simultaneously achieving high flexural strength and large deformation capacity. The steel-fiber-reinforced polymer-modified concrete prepared in this study exhibited relatively high flexural strength, large deformation capacity, and sustained load-carrying capacity after cracking. The flexural strength, ultimate flexural strain, and flexural elastic modulus of each group are presented in Table 4, while the representative flexural stress–strain and load–displacement curves are shown in Figure 3.

First, the combined effects of polymer modification and steel fiber reinforcement increased the flexural strength of concrete. Under their respective curing conditions, the 28 d mean flexural strengths of ordinary concrete and polymer-modified concrete were 5.13 and 7.77 MPa, respectively, corresponding to a 51.46% increase after polymer modification. With the incorporation of 2% and 5% ultrashort ultrafine steel fibers, the 28 d flexural strengths reached 8.38 and 9.95 MPa, respectively. These values were 63.35% and 93.96% higher than that of ordinary concrete and 7.85% and 28.05% higher than that of polymer-modified concrete, respectively. Among the two steel fiber contents investigated in this study, the 5% group showed a greater increase in flexural strength than the 2% group.

Second, the polymer-modified concretes exhibited early-age flexural-strength development. The 3 d flexural strength of the polymer-modified concrete was 5.74 MPa, corresponding to 73.87% of its 28 d strength. The 3 d flexural strengths of the polymer-modified concretes containing 2% and 5% ultrashort ultrafine steel fibers were 5.34 and 5.87 MPa, respectively, reaching 63.72% and 58.99% of their corresponding 28 d strengths. Both values exceeded the 28 d flexural strength of ordinary concrete. According to the *Code for Urban Road and Transportation Engineering Projects* (GB 55011-2021) [39], the flexural strength of cement concrete pavements subjected to very heavy or heavy traffic shall not be lower than 5 MPa. The polymer-modified concretes investigated in this study already met this strength requirement at 3 d. These early-age flexural strengths indicate that, from the perspective of mechanical performance, the material has potential for shortened curing periods and rapid reopening to traffic.

Third, polymer modification and steel fiber incorporation increased the ultimate flexural strain of the concrete. The mean ultimate flexural strain of ordinary concrete was only 178 με, whereas polymer modification increased this value to 1077 με, corresponding to an increase of 505.06%. With further incorporation of steel fibers, the ultimate flexural strains of the polymer-modified concretes containing 2% and 5% steel fibers reached 1286 and 1849 με, respectively. These values were 622.47% and 938.76% higher than that of ordinary concrete and 19.41% and 71.68% higher than that of polymer-modified concrete, respectively. Similarly to the trend observed for flexural strength, the 5% group showed a greater increase in ultimate flexural strain than the 2% group, indicating that, under the present test conditions, the higher of the two steel fiber contents investigated produced a greater improvement in deformation capacity.

Fourth, polymer incorporation reduced the flexural elastic modulus of concrete. The flexural elastic modulus of ordinary concrete was 33.74 GPa and decreased to 12.77 GPa after polymer modification. The flexural elastic moduli of the polymer-modified concretes containing 2% and 5% ultrashort ultrafine steel fibers were 13.34 and 15.64 GPa, respectively, representing reductions of 60.46% and 53.64% relative to ordinary concrete. The incorporation of steel fibers partially restored the stiffness of the composite, although the values remained lower than that of ordinary concrete. As shown by the stress–strain curves in Figure 3a, the polymer-modified concretes exhibited lower initial slopes. The lower flexural elastic modulus enabled greater deformation for a given increase in flexural stress and helped alleviate stress concentrations induced by temperature variations, shrinkage, and restraint from the underlying layer. In the context of the measured reduction in flexural elastic modulus, the flexible polymer network formed within the concrete reduced the overall stiffness of the cementitious matrix and contributed to alleviating stress concentrations at the aggregate–paste interfaces and crack tips. Consequently, the material retained relatively high flexural strength while providing a greater deformation reserve, resulting in a transition from high-stiffness brittle failure toward lower-modulus, tougher failure behavior.

**Figure 4:**
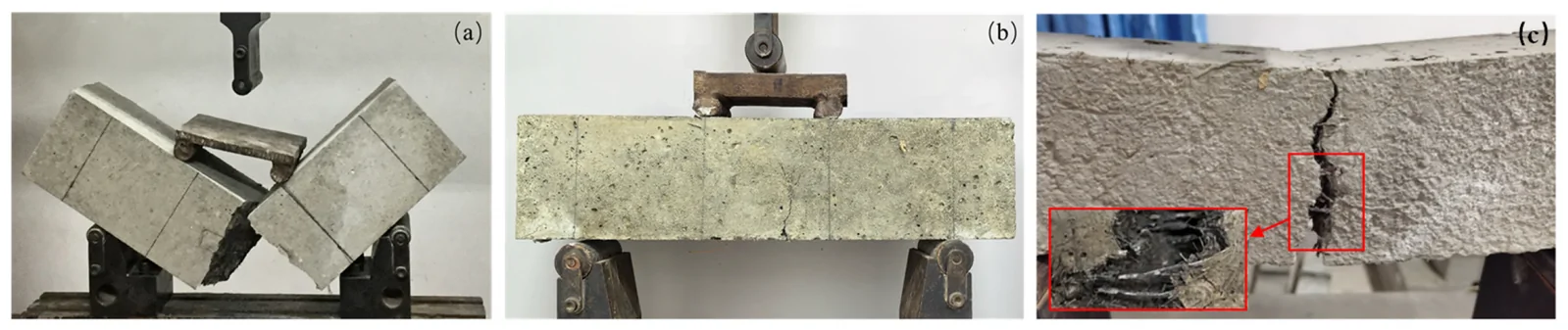
(**a**) Ordinary concrete; (**b**) polymer-modified concrete; (**c**) steel-fiber-reinforced polymer-modified concrete.

Fifth, the flexural load–displacement curves in Figure 3b reflect the post-cracking load-carrying characteristics of the steel-fiber-reinforced polymer-modified concrete. After reaching its flexural strength, ordinary concrete exhibited an almost vertical post-peak drop and failed in a brittle manner, as shown in Figure 4a. Although the polymer-modified concrete did not undergo brittle fracture after the peak load, as shown in Figure 4b, it still lost its load-carrying capacity rapidly in the post-peak stage. After steel-fiber incorporation, however, the specimens did not lose their load-carrying capacity immediately after cracking, regardless of fiber content. Instead, all load–displacement curves exhibited an extended post-peak descending branch with varying degrees of serrated fluctuation.

These fluctuations are associated with the progressive debonding, sliding, and pull-out of fibers during crack propagation. When local fibers were pulled out or underwent interfacial sliding, the load-carrying capacity temporarily decreased; the load was then redistributed to other fibers that had not yet been pulled out and to the surrounding matrix, allowing the specimen to continue carrying load. This repeated process of load reduction and redistribution delayed the development of the main crack through the specimen and the onset of complete fracture, as shown in Figure 4c, thereby providing the material with sustained post-cracking load-carrying capacity.

Overall, the polymer primarily improved the deformation compatibility of the matrix and interfacial integrity, whereas the steel fibers mainly contributed through post-cracking crack bridging and energy dissipation during fiber pull-out. These two components played different but complementary roles in the composite, enabling the steel-fiber-reinforced polymer-modified concrete to exhibit relatively high flexural strength, large ultimate flexural strain, and sustained post-cracking load-carrying capacity.

### 3.2. Compressive Strength and Compressive Failure Mode

**Figure 5:**
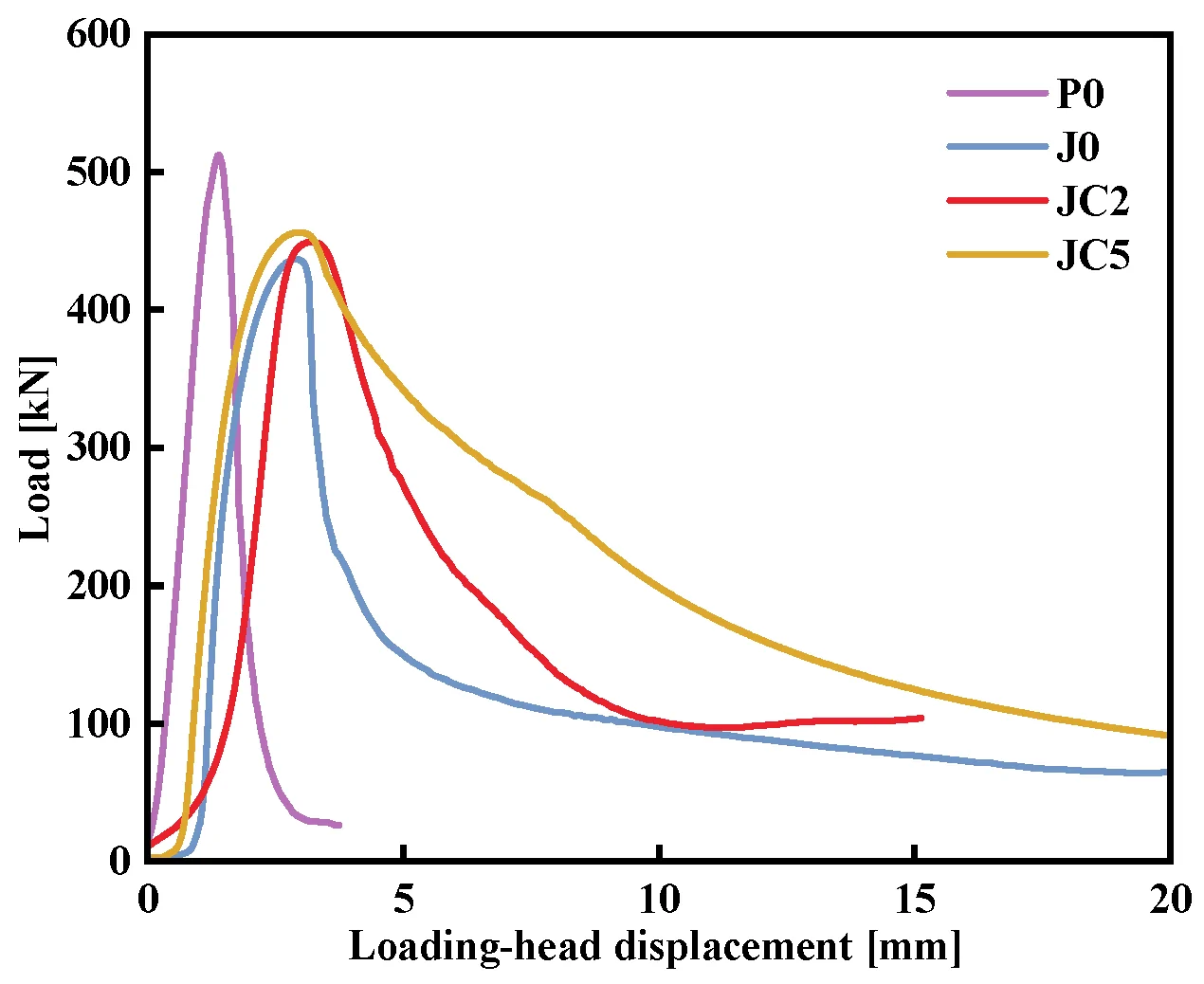
Compressive load–displacement curves.

Although polymer modification can improve the deformation capacity and toughness of concrete, it is often accompanied by a reduction in the compressive strength of the matrix. For pavement materials, once the design load-bearing requirements are satisfied, further increasing compressive strength is generally not the primary performance objective [40,41]. The steel-fiber-reinforced polymer-modified concrete prepared in this study exhibited relatively higher compressive strength while maintaining large deformation capacity and toughness. In addition, its compressive failure mode changed from sudden brittle failure to a more ductile mode characterized by larger deformation and post-cracking load-carrying capacity. The compressive strengths and corresponding load–displacement curves of the different groups are presented in Table 5 and Figure 5, respectively.

First, the compressive strength of the polymer-modified concrete was 42.47 MPa. With the incorporation of 2% and 5% ultrashort ultrafine steel fibers, the compressive strength increased to 43.07 and 45.13 MPa, corresponding to increases of 1.41% and 6.26%, respectively. The steel fibers restrained lateral deformation during compression and bridged microcracks within the matrix, thereby delaying crack propagation and partially compensating for the reduction in compressive strength associated with polymer modification. However, because the compressive strength of concrete is governed primarily by the combined contribution of the aggregate and cementitious matrix, the overall increase in peak compressive strength provided by the steel fibers remained limited.

Second, all steel-fiber-reinforced polymer-modified concretes exhibited early-strength development. The 3 d compressive strength of the polymer-modified concrete reached 49.37% of its 28 d strength. After the incorporation of 2% and 5% ultrashort ultrafine steel fibers, the corresponding ratios increased to 54.40% and 53.78%, respectively. The filling and film-forming effects of the polymer in pores and interfacial regions, together with the restraint provided by steel fibers against early microcracking, contributed to the development of a stable load-bearing system at an early age. This early-age strength development suggests that the material has potential for use in engineering applications requiring early reopening to traffic, such as bridge-deck paving and rapid road repair.

**Figure 6:**
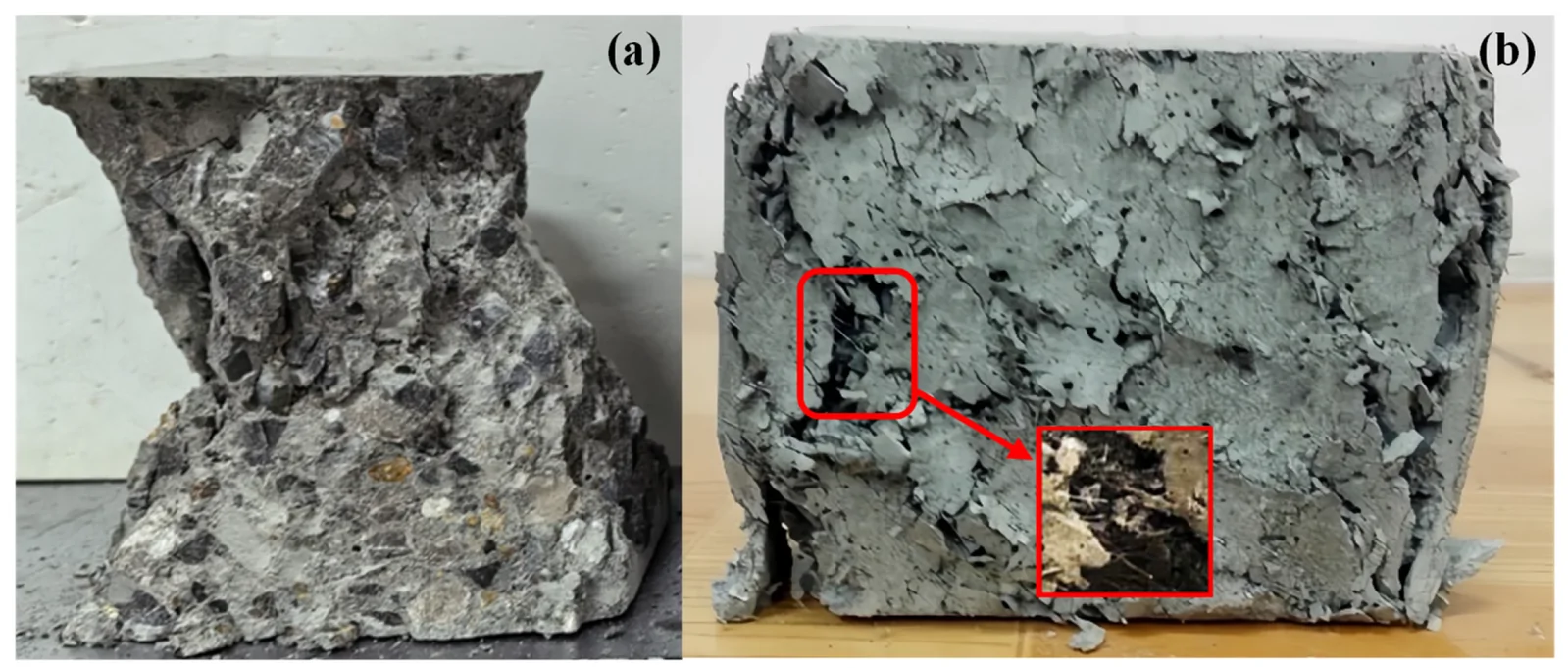
Compressive failure modes: (**a**) ordinary concrete; (**b**) steel-fiber-reinforced polymer-modified concrete.

Third, the combined effects of the polymer and steel fibers were reflected mainly in the post-peak toughness and failure mode. As shown by the compressive load–displacement curves in Figure 5, ordinary concrete exhibited brittle spalling and disintegration after reaching the peak load, as shown in Figure 6a. In contrast, the descending branches of the load–displacement curves for the steel-fiber-reinforced polymer-modified concrete were extended, and the rate of post-peak load decay was reduced. The specimens also retained a certain residual load-carrying capacity at relatively large displacements. The steel fibers continued to transfer load and dissipate energy through bridging, sliding, and pull-out, while the polymer improved the integrity of the fiber–matrix interface and the deformation compatibility of the composite, thereby delaying the formation of through-cracks. As a result, the steel-fiber-reinforced polymer-modified concrete exhibited relatively large compressive deformation at failure, as shown in Figure 6b.

Overall, steel-fiber-reinforced polymer modification produced only a limited increase in peak compressive strength, but it provided both early-age load-bearing capacity and post-peak load-carrying capacity after cracking, changing the failure behavior of concrete from rapid brittle failure to a more ductile failure mode.

### 3.3. Impact Toughness and Crack Propagation

**Figure 7:**
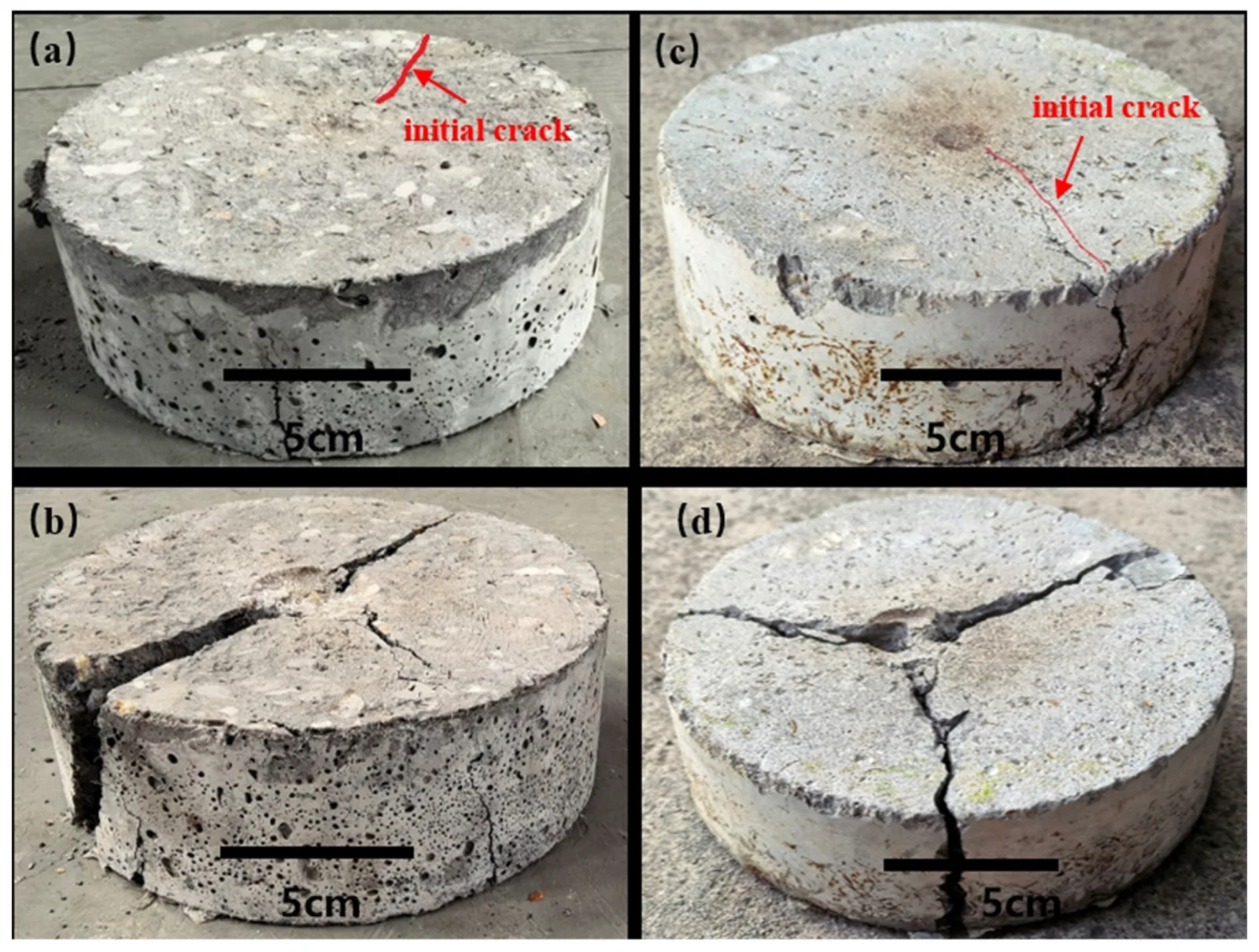
Representative failure patterns in the drop-weight impact test: (**a**,**b**) polymer-modified concrete; (**c**,**d**) steel-fiber-reinforced polymer-modified concrete.

Pavements are subjected to repeated impact loading over their service life due to high-speed vehicle passage, emergency braking, and heavy traffic. Conventional concrete pavement materials are prone to brittle cracking, which can rapidly develop into fragmentation failure [22,42]. Therefore, improving repeated-impact resistance and sustained post-cracking load-carrying capacity is particularly important for pavement materials. The steel-fiber-reinforced polymer-modified concrete prepared in this study was able to delay the initiation and propagation of impact-induced cracks and maintained relatively good integrity and sustained load-carrying capacity after cracking. The drop-weight impact test results for the different concrete groups are presented in Table 6, and representative failure patterns at initial cracking and final failure are shown in Figure 7.

First, polymer incorporation improved the impact resistance of concrete. The mean numbers of impacts at initial cracking and failure for the polymer-modified concrete were 4.67 and 18.33, respectively, representing increases of approximately 251.12% and 997.60% relative to ordinary concrete. The corresponding nominal impact energies at initial cracking and failure were 103.01 and 404.66 J, respectively. Meanwhile, the post-cracking impact reserve Δ*N* of the polymer-modified concrete reached 13.67 impacts, corresponding to a post-cracking nominal impact energy reserve *ΔW* of 301.66 J, which was 3998.64% higher than that of ordinary concrete. These results indicate that, under the same impact conditions, polymer-modified concrete required a greater cumulative nominal energy input to reach both the first visible crack and final failure and retained a higher resistance to repeated impacts after initial cracking.

Second, the incorporation of steel fibers further improved the impact resistance of the polymer-modified concrete, and the increase was greater at a steel-fiber content of 5% than at 2%. For the polymer-modified concrete containing 2% ultrashort ultrafine steel fibers, the mean numbers of impacts at initial cracking and failure were 8.67 and 36.00, respectively. These values were 551.87% and 2055.69% higher than those of ordinary concrete and 85.65% and 96.40% higher than those of polymer-modified concrete, respectively. When the steel-fiber content was increased to 5%, the corresponding mean numbers of impacts reached 55.67 and 146.67, representing increases of 4085.71% and 8682.63% relative to ordinary concrete and 1092.07% and 700.16% relative to polymer-modified concrete, respectively. The corresponding nominal impact energies at initial cracking and failure followed the same trends as the impact numbers.

In addition, the ΔN values, and hence the corresponding ΔW values, of the polymer-modified concretes containing 2% and 5% steel fibers were 2.00 and 6.66 times those of the polymer-modified concrete without steel fibers, respectively. This further indicates that steel-fiber incorporation prolonged the repeated-impact process from initial cracking to final failure. A direct comparison between the two fiber contents showed that the mean numbers of impacts at initial cracking, at failure, and from initial cracking to failure for the 5% group were 6.42, 4.07, and 3.33 times those of the 2% group, respectively. Within the fiber-content range investigated in this study, the higher fiber content of the 5% group resulted in a greater number of fibers within the same matrix volume, reducing the average fiber spacing and promoting the formation of a denser crack-bridging and crack-arresting network. This provided stronger restraint against crack initiation and propagation and delayed the formation of through-cracks.

Third, the representative impact failure patterns of the polymer-modified concrete and steel-fiber-reinforced polymer-modified concrete are shown in Figure 7. Ordinary concrete developed a through-crack almost immediately after the first impact and rapidly split into two parts, exhibiting a typical brittle impact failure mode. At initial cracking, the polymer-modified concrete showed only a short, fine crack extending outward from the impact center; the crack remained narrow and had not yet propagated through the specimen, which therefore retained its overall integrity, as shown in Figure 7a. With increasing numbers of impacts, the initial crack progressively propagated and widened to form the main crack governing failure, accompanied by the initiation and growth of secondary cracks. The polymer-modified concrete underwent a relatively prolonged repeated-impact process from initial cracking to final failure, indicating that polymer modification delayed the unstable propagation of cracks into through-cracks, as shown in Figure 7b.

At initial cracking, the ultrashort ultrafine steel-fiber-reinforced polymer-modified concrete exhibited only fine and short local cracks in the vicinity of the impact center. Both the crack propagation range and crack opening were restrained, and the specimen maintained good overall integrity, as shown in Figure 7c. At impact failure, multiple radial cracks extended outward from the impact center. As the main crack propagated, crack branching and the initiation and continued growth of new cracks also occurred. The cracks remained constrained by the bridging action of the steel fibers, and the specimen did not separate completely, as shown in Figure 7d.

A comparison across the different materials shows that polymer modification changed the failure mode of ordinary concrete from instantaneous brittle splitting after a very small number of impacts to a process governed by the gradual propagation of multiple cracks. The combined modification by polymer and ultrashort ultrafine steel fibers further transformed the unstable failure controlled by a single through-crack into a progressive, more ductile failure process governed by the initiation, branching, and stable propagation of multiple cracks. After the formation of macroscopic cracks, the material was still able to maintain its integrity and residual load-carrying capacity through the deformation compatibility provided by the polymer network and the crack-bridging action of the steel fibers, thereby exhibiting sustained post-cracking load-carrying capacity. As a result, the failure mode shifted from brittle impact fracture to a more ductile failure mode.

### 3.4. SEM Analysis

**Figure 8:**
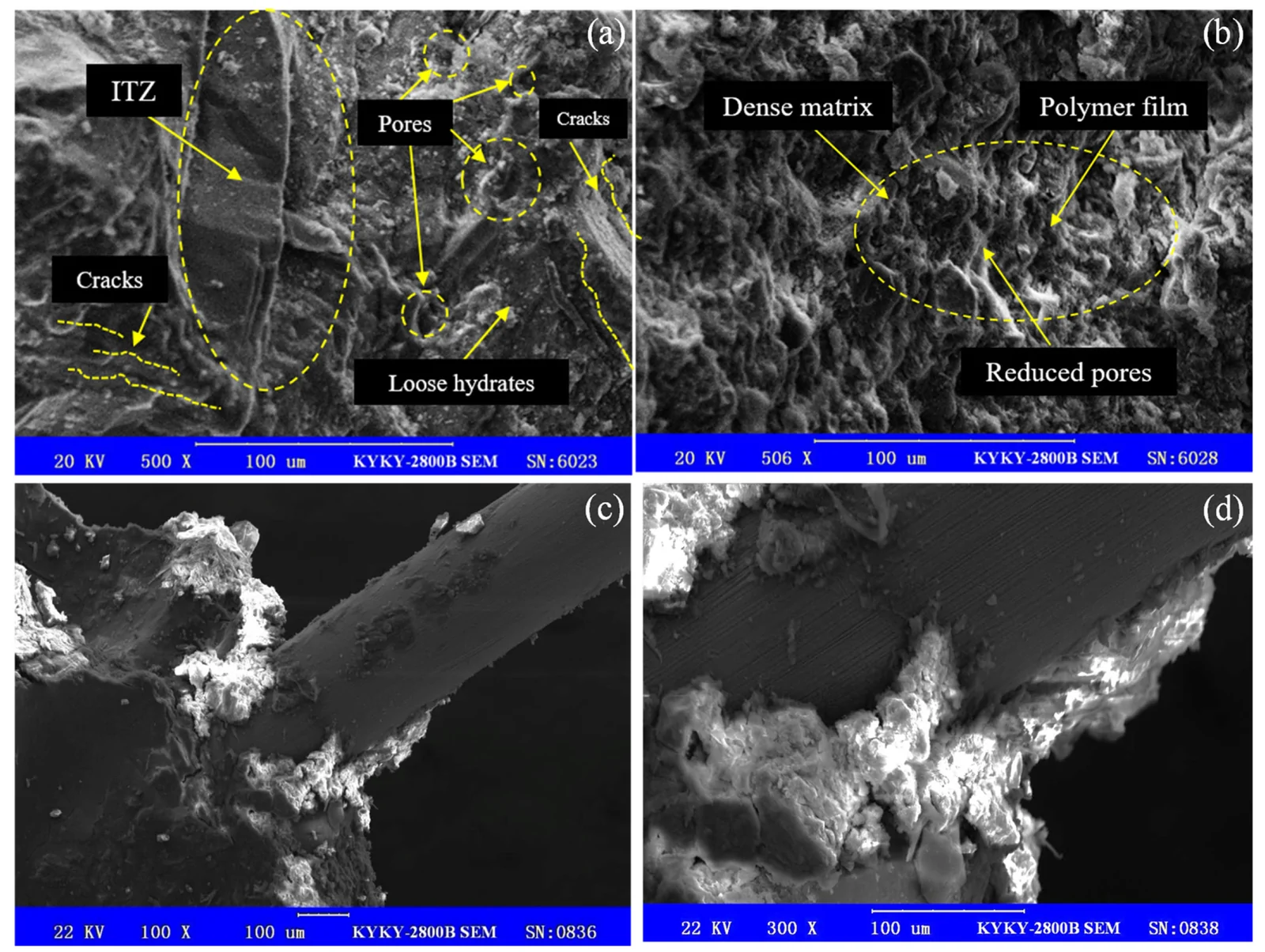
SEM images: (**a**) ordinary concrete at 500× magnification; (**b**) polymer-modified concrete at 500× magnification; (**c**) steel-fiber-reinforced polymer-modified concrete at 100× magnification; (**d**) steel-fiber-reinforced polymer-modified concrete at 300× magnification.

The microstructural morphology of ordinary concrete and the characteristics of the steel fiber–matrix interface are shown in Figure 8. In Figure 8a, the hydration products in ordinary concrete appear loosely interconnected, with numerous pores and microcracks and a distinguishable interfacial transition zone (ITZ). In Figure 8b, continuous film-like structures are observed on the surfaces of the hydration products in the polymer-modified concrete and interconnect the dispersed hydration products. Some pores and microcracks are filled, and no distinct ITZ is observed. Compared with ordinary concrete, the polymer-modified concrete therefore exhibits a more continuous and compact microstructural morphology.

As shown in Figure 8c, considerable polymer–hydration-product residues remain attached to the surface of the pulled-out steel fiber, suggesting that fiber pull-out did not occur solely through debonding along the fiber–matrix interface but was also accompanied by local detachment of the interfacial layer and adjacent matrix. This morphological feature suggests that the presence of the polymer contributes to a more integrated fiber–matrix interface, thereby helping to maintain load transfer during fiber pull-out and facilitate crack bridging. In Figure 8d, continuous hydration products and film-like substances are observed around the root of the steel fiber, with no distinct annular gap visible at the interface.

By considering the microstructural morphologies observed by SEM together with the macroscopic mechanical responses, the strengthening and toughening behavior of the steel-fiber-reinforced polymer-modified concrete can be interpreted as follows. The pore filling, continuous film-like structures, and more continuous interfacial morphologies observed after polymer modification support the interpretation that the polymer improves the integrity of the matrix and interfacial regions and enhances deformation compatibility. This interpretation is consistent with the larger ultimate flexural strain and the different failure characteristics of the polymer-modified concrete. After steel-fiber incorporation, the macroscopic crack patterns and flexural load–displacement responses showed sustained post-cracking load-carrying capacity. Together with the fiber pull-out and surface residues observed by SEM, these results support the interpretation that steel fibers contribute to post-cracking load transfer and energy dissipation through crack bridging, interfacial debonding, sliding, and pull-out. Overall, the matrix and interfacial morphological changes associated with polymer modification, together with the post-cracking bridging action of the steel fibers, provide a consistent microstructural interpretation of the larger deformation capacity, extended post-peak response, and greater repeated-impact resistance observed in the mechanical tests.

## 4. Conclusions

(1) Polymer modification and ultrashort ultrafine steel fibers played distinct but complementary roles in the composite. Polymer modification primarily reduced the flexural stiffness and improved the deformation compatibility of the matrix. Under their respective curing regimes, the ultimate flexural strain of polymer-modified concrete was 505.06% higher than that of ordinary concrete. With further incorporation of steel fibers, both flexural load-carrying capacity and deformation capacity increased. In particular, the addition of 5% steel fibers increased the 28 d flexural strength and ultimate flexural strain of the polymer-modified concrete by 28.05% and 71.68%, respectively. These results indicate that the contribution of steel fibers lies not only in increasing peak load-carrying capacity, but also in enhancing load transfer and deformation capacity after crack formation.
(2) Ultrashort ultrafine steel fibers altered the post-cracking load-carrying behavior of the material. The load-carrying capacity of polymer-modified concrete decreased rapidly after the peak load, whereas the steel-fiber-reinforced specimens exhibited a longer post-peak descending response and retained residual load-carrying capacity at larger deformations. Crack bridging by the fibers, followed by debonding, sliding, and pull-out, delayed the formation of a through-crack, changing the failure response from rapid post-peak load loss to progressive failure with sustained post-cracking load-carrying capacity.
(3) The repeated-impact resistance of the steel-fiber-reinforced polymer-modified concrete was reflected not only in delayed initial cracking, but also in an increased damage tolerance from initial cracking to final failure. For the 5% steel-fiber group, both the number of impacts to failure and the nominal impact energy at failure were 700.16% higher than those of the polymer-modified concrete. Its post-cracking impact reserve *ΔN* and post-cracking nominal impact energy reserve *ΔW* were 6.66 times those of the polymer-modified concrete. These results indicate that, after the formation of macroscopic cracks, the material could still withstand multiple repeated impacts while maintaining relatively good integrity and sustained load-carrying capacity.
(4) The SEM observations were consistent with the macroscopic mechanical responses. After polymer modification, continuous film-like structures and more continuous matrix and interfacial morphologies were observed, while considerable residual material remained attached to the surfaces of pulled-out steel fibers. These microstructural features support the interpretation that the polymer improves deformation compatibility of the matrix and interfacial integrity, whereas the steel fibers contribute to post-cracking load transfer through crack bridging and pull-out.
(5) These performance characteristics provide a material-design basis for addressing premature cracking, brittle failure, insufficient post-cracking load-carrying capacity, and rapid damage accumulation under repeated loading in specialized pavement materials. The combination of relatively high flexural load-carrying capacity, large deformation capacity, sustained post-cracking load-carrying capacity, and high impact damage tolerance indicates good application potential in engineering scenarios requiring high load capacity, crack control, and damage tolerance, such as heavy-traffic pavements, steel bridge-deck pavements, and rapid repair.
(6) Future research should further clarify the relationships among material composition, microstructure, and long-term performance. First, additional steel fiber contents between 2% and 5% should be investigated, together with a larger number of replicate specimens and characterization of fiber dispersion and orientation, to establish a more reliable fiber-content–performance relationship. Second, the fiber–matrix interfacial behavior should be examined in greater depth through quantitative characterization of pore structure and the interfacial transition zone, as well as single-fiber pull-out tests. Further studies are also needed on flexural fatigue and long-term durability under temperature, moisture, and other environmental actions. Full-scale pavement or steel bridge-deck paving tests, together with in-service field monitoring, should be conducted to further evaluate the long-term performance and applicability of the material under actual engineering conditions. In addition, the mechanical responses of ordinary concrete and polymer-modified concrete under different curing regimes should be systematically compared to clarify the influence of curing conditions on performance comparisons between the two materials.

## Figures and Tables

**Table 1:** Type and main physical and mechanical properties of the steel fiber.

Steel Fiber Type	Length [mm]	Diameter [mm]	Aspect Ratio	Density [t/m^3^]	Elastic Modulus [GPa]	Tensile Strength [MPa]
Melt-extracted ultrashort ultrafine steel fiber	6	0.20	30	7.80	240	880

**Table 2:** Sieve analysis results of the yellow sand.

Sieve Size [mm]	9.50	4.75	2.36	1.18	0.60	0.30	0.150	0.075
Passing percentage [%]	100	98.20	71.40	39.90	16.90	7.80	1.50	0.70

**Table 3:** Mix proportions investigated in this study.

No.	Mix ID	Steel Fiber Type	Steel Fiber Content [%]
1	P0	-	0
2	J0	-	0
3	JC2	Ultrashort ultrafine steel fiber	2
4	JC5	Ultrashort ultrafine steel fiber	5

**Table 4:** Flexural test results (mean ± standard deviation, n = 3).

Group	Flexural Strength at 3 d [MPa]	Flexural Strength at 7 d [MPa]	Flexural Strength at 28 d [MPa]	Ultimate Flexural Strain at 28 d [με]	Flexural Elastic Modulus at 28 d [GPa]
P0	3.94 ± 0.25	4.75 ± 0.31	5.13 ± 0.44	178 ± 42	33.74 ± 1.22
J0	5.74 ± 0.23	7.48 ± 0.42	7.77 ± 0.43	1077 ± 60	12.77 ± 1.71
JC2	5.34 ± 0.45	7.81 ± 0.34	8.38 ± 0.43	1286 ± 195	13.34 ± 0.55
JC5	5.87 ± 0.24	7.95 ± 0.18	9.95 ± 0.67	1849 ± 179	15.64 ± 1.07

**Table 5:** Compressive strength results (mean ± standard deviation, n = 3).

Group	Compressive Strength at 3 d [MPa]	Compressive Strength at 7 d [MPa]	Compressive Strength at 28 d [MPa]
P0	13.13 ± 1.62	22.47 ± 2.73	46.97 ± 6.65
J0	20.97 ± 1.53	37.57 ± 2.98	42.47 ± 3.90
JC2	23.43 ± 1.31	34.20 ± 2.17	43.07 ± 1.68
JC5	24.27 ± 1.25	35.77 ± 2.89	45.13 ± 0.40

**Table 6:** Drop-weight impact test results (five specimens tested per group; mean ± standard deviation calculated from the three retained values after excluding the maximum and minimum values).

Group	Impact Count at First Cracking, *N*_1_	Nominal Impact Energy at First Cracking *W*_1_ [J]	Impact Count at Final Failure, *N*_2_	Nominal Impact Energy at Final Failure, *W*_2_ [J]	Post-Cracking Impact Reserve, *ΔN*	Nominal Post-Cracking Impact-Energy Reserve, *ΔW* [J]
P0	1.33 ± 0.58	29.43 ± 12.74	1.67 ± 0.58	36.79 ± 12.74	0.33 ± 0.58	7.36 ± 12.74
J0	4.67 ± 0.58	103.01 ± 12.74	18.33 ± 2.52	404.66 ± 55.55	13.67 ± 2.52	301.66 ± 55.55
JC2	8.67 ± 3.06	191.30 ± 67.43	36.00 ± 7.21	794.61 ± 159.17	27.33 ± 9.02	603.32 ± 199.06
JC5	55.67 ± 12.66	1228.70 ± 279.49	146.67 ± 17.79	3237.30 ± 392.58	91.00 ± 21.63	2008.60 ± 477.50

## Data Availability

The raw data supporting the conclusions of this article will be made available by the authors on request.

## Nomenclature

Abbreviations, symbols and specimen codes.

**Abbreviation/code**	**Definition**	
SEM	Scanning electron microscopy	
ITZ	Interfacial transition zone	
P	Ordinary concrete	
J	Polymer-modified concrete	
C	Ultrashort ultrafine steel fiber	
P0	Ordinary concrete without polymer or steel fibers	
J0	Polymer-modified concrete without steel fiber	
JC2	Polymer-modified concrete containing 2 vol.% ultrashort ultrafine steel fiber	
JC5	Polymer-modified concrete containing 5 vol.% ultrashort ultrafine steel fiber	
Symbol	Definition	Unit
$N_{1}$	Cumulative number of impacts at first visible cracking	-
$N_{2}$	Cumulative number of impacts at final failure	-
∆N	Post-cracking impact reserve, $∆N=N_{2}-N_{1}$	-
$W_{1}$	Nominal impact energy at first cracking	J
$W_{2}$	Nominal impact energy at final failure	J
∆W	Nominal post-cracking impact-energy reserve	J
*m*	Drop-hammer mass	kg
*g*	Gravitational acceleration	$m/s^{2}$
*n*	Number of independent replicate specimens	-
*h*	Drop height in the repeated drop-weight impact test	mm
